# The Effects of Previous Error and Success in Alzheimer’s Disease and Mild Cognitive Impairment

**DOI:** 10.1038/s41598-019-56625-2

**Published:** 2019-12-27

**Authors:** T. J. Crawford, S. Taylor, D. Mardanbegi, M. Polden, T. W. Wilcockson, R. Killick, P. Sawyer, H. Gellersen, I. Leroi

**Affiliations:** 1 Psychology Department, Lancaster University, Centre for Ageing Research, Lancaster, LA1 4YF UK; 2 Department of Statistics, Lancaster University, Fylde College, Lancaster, LA1 4YF UK; 3 Computing and Communications Department, Lancaster University, Lancaster, UK; 4 School of Sport, Exercise and Health Sciences, Loughborough University, Loughborough, UK; 5 Engineering and Applied Science, Aston University, Birmingham, UK; 6 Global Brain Health Institute, Trinity College Dublin, Dublin, Ireland

**Keywords:** Risk factors, Psychiatric disorders

## Abstract

This work investigated in Alzheimer’s disease dementia (AD), whether the probability of making an error on a task (or a correct response) was influenced by the outcome of the previous trials. We used the antisaccade task (AST) as a model task given the emerging consensus that it provides a promising sensitive and early biological test of cognitive impairment in AD. It can be employed equally well in healthy young and old adults, and in clinical populations. This study examined eye-movements in a sample of 202 participants (42 with dementia due to AD; 65 with mild cognitive impairment (MCI); 95 control participants). The findings revealed an overall increase in the frequency of AST errors in AD and MCI compared to the control group, as predicted. The errors on the current trial increased in proportion to the number of consecutive errors on the previous trials. Interestingly, the probability of errors was reduced on the trials that followed a previously corrected error, compared to the trials where the error remained uncorrected, revealing a level of adaptive control in participants with MCI or AD dementia. There was an earlier peak in the AST distribution of the saccadic reaction times for the inhibitory errors in comparison to the correct saccades. These findings revealed that the inhibitory errors of the past have a negative effect on the future performance of healthy adults as well as people with a neurodegenerative cognitive impairment.

## Introduction

How do we stop ourselves doing something that we know we should not be doing, and how do our previous errors influence our current behaviour? A convenient way of addressing these questions in the laboratory employs the antisaccade task^1^ (AST). There is growing interest in the potential of the paradigm as a measure of inhibitory control in healthy and clinical populations. If we can uncover the major underlying factors that give rise to these errors of control in the AST, we will be in a better position to understand how these errors might be mitigated in various clinical and non-clinical populations to improve cognitive control. The AST presents a stimulus to the left or right of the screen whilst the observer is required to avoid the natural impulse to look towards the stimulus, and instead to look away, to the opposite side of the screen. Healthy adults fail to inhibit the impulse to look towards the target on approximately 20% of trials^2^. The failure rate is substantially higher in AD (55–75%)^3,4^. In healthy participants, the error is usually followed shortly afterwards by a self-correcting saccadic eye movement, whereas these corrections occur infrequently in AD dementia, and then only after a substantial delay. The frequency is positively correlated with the severity of the dementia and memory impairment^3,5^. To date, no study has attempted to track the moment-by-moment changes in cognitive control in AD dementia during the AST. Could there be a mechanism that resets or reboots after each error so that the system starts afresh on the current trial? Or is the outcome of the current trial subject to the influence of the past events? A simple way forward would be to examine the effect of past trials on the present trial performance. In this context, we investigated the extent to which the AST errors might be influenced by the outcomes of the previous trials. Tatler and Hutton^6^ conducted the only study to examine the effects of historical antisaccade trials on the current trial in a sample of young healthy participants. This work revealed that an inhibition error on a preceding trial was strongly associated with an increase in the probability of an error on the current trial. Interestingly, the effects were not limited to the immediately preceding trial. They found a linear and cumulative effect of the number of past error trials on the probability of an error occurring on the current trial. It is not known whether this error phenomenon is also found within a clinical cohort.

Several influential models of cognitive control have proposed that behavioural errors are detected on-line, by neural monitors (such as the Anterior Cingulate cortex) and used to adjust and correct performance on a moment-by-moment basis. According to Dehaene and colleagues^7–9^, the on-line control by neural and behavioural error detection and correction networks (i.e. adaptive networks) operates primarily in the context of the conscious awareness of the task conflict. However, according to Miller and Cohen^10^ the neural network that includes the Anterior cingulate – Dorsolateral Prefrontal Cortex projections, is activated by the task conflict that is detected with or without conscious awareness. Tatler and Hutton^6^ found, using the AST, that the error rates in fact increased, rather than decreased, on the trials that followed an error on the previous trial. This seemed to provide little support for either of these cognitive control models. They concluded that “…an increased probability of making an error following an error on the previous trial is opposite to the predictions made by the popular conflict monitoring account of antisaccade performance”p394. However, a key prediction of these models relates to the issue of whether or not the conflicts and errors are detected, whether consciously or unconsciously. As mentioned by Tatler and Hutton^6^, within a healthy (young) population there is little variability in the detection of the errors and conflict; error correction in the AST is close to 100%. Thus, young healthy participants typically show high error detection rates, as revealed by the very high proportion of corrective saccades. Therefore, whilst it is surprising that AST errors increased following a previous error, their analysis was not a critical or devastating test of the models. A more critical test for the models would contrast the effects of the trials in which the errors are detected, with the trials where the errors are not successfully detected and corrected. The cognitive control models would predict that the errors that are detected and corrected (either consciously or unconsciously according to Miller and Cohen^10^) should lead to a lower probability of errors on the following trial, in comparison to the errors that are not detected and corrected. This prediction can be best examined using participants with Alzheimer’s Disease dementia and MCI, as they are known to generate a relatively high proportion of both corrected and uncorrected errors. This prediction cannot be easily examined with healthy control participants who produce relatively few uncorrected errors in the AST.

Therefore, the following questions were addressed in this study: Do the past errors and successes have an effect on AD performance on the current trial? Are the inhibitory errors mediated by the speed of the reaction time on the current trial^11^? Answers to these questions will provide a more comprehensive insight into inhibitory control as a candidate biomarker for MCI/AD and in the monitoring of the progression of the disease.

## Results

The mean and standard deviation of the participant demographic and cognitive characteristics are presented in Table 1. The groups did not differ with regards to gender (chi-squared test, χ^2^ = 2.400, p = 0.301, Cramer’s V = 0.077), but did differ on mean age (ANOVA, F(2,199) = 13.469, p < 0.01). As expected the dementia scores on MOCA differed between the groups (ANOVA, F(2,194) = 63.715, p < 0.001); where the AD group had lower dementia scores on the MOCA, compared to the controls & MCI; the MCI group had lower scores than the controls: Table 1 also reveals that the AD and MCI groups had lower scores than the control group on reverse digit span, and reverse and forwards spatial span. The AD and MCI groups did not differ significantly on the reverse digit or the spatial span tasks.

**Table 1:** Participant demographic and cognitive characteristics.

		Control	MCI	AD	ANOVA
Sex	Male	36	31	21	
	Female	59	34	21	
Age	Mean ± SD	66.7 ± 8.6	70.5 ± 8.0	74.4 ± 7.8	F: 13.47, df: (2,199), p: <0.01, η^2^: 0.135
	Range	48–90	56–86	57–90	
MOCA	Mean ± SD	27.2 ± 3.2	22.9 ± 4.4	19.7 ± 5.9	F: 20.04, df: (2,195), p: <0.01, η^2^: 0.256
Digit Span-F	Mean ± SD	10.7 ± 2.5	10.2 ± 2.4	10.1 ± 2.4	F: 1.06, df: (2,192), p: 0.349, η^2^: 0.011
Digit Span-R	Mean ± SD	7.2 ± 2.7	6.2 ± 2.5	5.5 ± 2.3	F: 4.88, df: (2,192), p: <0.01, η^2^: 0.051
Spat Span-F	Mean ± SD	7.5 ± 1.4	6.7 ± 1.6	5.8 ± 1.7	F: 11.16, df: (2,180), p: <0.01, η^2^: 0.124
Spat Span-R	Mean ± SD	6.9 ± 1.7	5.9 ± 1.9	5.5 ± 2.1	F: 4.89, df: (2,180), p: <0.01, η^2^: 0.054

MOCA - Montreal Cognitive Assessment for Dementia; Digit Span-F – Digit Memory Span forwards assessment score; Digit Span-R – Digit Memory Span reverse assessment score; Spatial Span-F – Spatial Memory Span forwards assessment score; Spatial Span- R – Spatial Memory Span reverse assessment score.

### Antisaccade task completion analyses

One hundred and sixty participants performed the complete sequence of 24 trials. Others had shorter sequences with no lower than 11 trials. These participants were balanced across the groups (chi-squared test, χ^2^ = 1.778, p = 0.411, Cramer’s V = 0.066). This resulted in an overall total of 4732 out of a possible 4848 trials, whereby the frequency of incomplete sequences was independent of the cognitive groups (chi-squared test, χ^2^ = 4.639, p = 0.098, Cramer’s V = 0.022). 79.2% of participants performed the full set of 24 trials and more than 95% of participants completed over 20 trials. We conducted a complete case -analysis (using only the 160 participants with the full 24 trials) and found no substantial differences in the overall pattern of the effects.

### Are antisaccade errors increased in the AD and MCI groups compared to the controls

**Figure 1:**
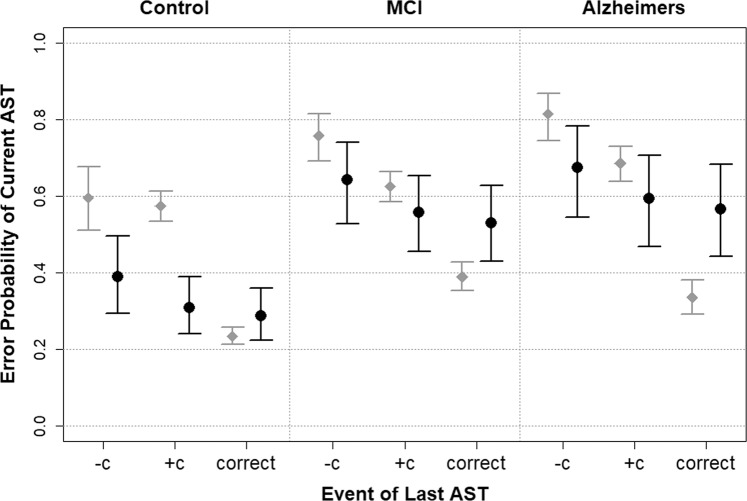
Graph showing the mean error probability on the current trial, after accounting for random subject variability in relation to the outcomes on the previous trial. The sample estimates (in grey with diamond points) do not account for subject variation and so there is over-confidence in the probability estimates. The estimates derived from the model (black with circle points) do account for subject variation. Despite the differences in the error probability estimates, the broad and expected patterns between groups is preserved as well as the dependence on the last (i.e. previous) AST. −c = the previous trial was an error with no correction; +c = the previous trial was an error with correction; correct = the previous trial was a correct antisaccade. Mild cognitive impairment (MCI) and Alzheimer’s disease (AD).

The antisaccade data can be classified into 3 types of response outcomes. **Correct:** a saccade is correctly directed into the opposite target hemifield; **Error + c:** an incorrect saccade directed towards the target, together with a later corrective saccade away from the target into the opposite hemifield; **Error − c:** an error with no corrective saccade component. Figure 1 shows the proportion of **Correct** responses; errors followed by a correction (**Error** + **c):** and errors with no correction (**Error** − **c**) by participant group. The AD and MCI participants have a significantly higher proportion of **Error** + **c** and **Error** − **c** responses on the antisaccade trials in comparison to the control group (chi-squared test, χ^2^ = 193.06, p < 0.001, Cramer’s V = 0.101). The distributions of the AD and MCI were overlapping, consistent with the view that the MCI can be considered as a preclinical AD group. There was a significant effect across groups for **Error** − **c** responses with respect to the proportion of error trials: (chi-squared test, χ^2^ = 25.477, p < 0.001, Cramer’s V = 0.077) and in relation to total number of observations (i.e. “Errors” + “Correct”): (chi-squared test, χ^2^ = 86.734, p < 0.001, Cramer’s V = 0.096).

### Does the probability of an error trial *increase* if the previous trial has been an error

Table 2 shows the mean antisaccade errors on the current trial in relation to the outcomes of the previous trial for each group. A similar pattern is evident in all three groups. The numerical values represent the number and proportion of antisaccade errors in each category, and the log-odds ratio of the patient groups relative to the control group for each of the previous trial outcome events (standard errors for each estimate are presented in parentheses). Trials where an inhibitory error occurred, irrespective of whether this was subsequently corrected, generated a higher than 50% chance of resulting in a further error on the current trial (e.g. For controls with last uncorrected error event, one-tailed Z-test, Z-statistic: 2.23, p = 0.013; see Table 2). Figure 1 shows the error probability in relation to the previous trial before, and after, accounting for the individual random variability. The sample estimates (in grey with diamond points) do not account for subject variation and so there is over-confidence in the probability estimates. The estimates derived from the model (black with circle points) represent the account for individual subject variation. Despite the differences in the error probability estimates, the broad and expected patterns between the groups is preserved as well as the dependence on the previous AST trial.

**Table 2:** The effect of the previous trial on the current trial.

Previous Trial	Control	MCI	AD
**Error (−c)**			
Errors (N)	78	141	123
Proportion (se)	59.5% (0.043)	75.8% (0.031)	81.5% (0.032)
Log-odds (se)	—	0.756 (0.259)	1.094 (0.286)
**Error (+c)**			
Errors (N)	362	374	262
Proportion (se)	57.4% (0.020)	62.5% (0.020)	68.6% (0.024)
Log-odds (se)	—	0.216 (0.121)	0.484 (0.140)
Correct			
Errors (N)	319	260	140
Proportion (se)	23.4% (0.011)	38.9% (0.019)	33.5% (0.023)
Log-odds (se)	—	0.737 (0.091)	0.502 (0.112)
**Error (−c) vs**. **Error (+c)**			
Log-odds (se)	0.089 (0.195)	0.629 (0.191)	0.699 (0.237)
Z-statistic (p-value)	0.458 (0.647)	3.297 (<0.001)	2.954 (0.003)

se – standard error.

### We also addressed the inverse question: Did the probability of an error response *decrease* on the current trial, if the previous trial was a correct response

Table 2 reveals that the probability of an error on the current trial was significantly below 50% (chance) if the immediately preceding trial was correct (c). The form of the effect is very similar for the Control, MCI and AD groups (Fig. 1). Correct antisaccade trials are less likely to be followed by trials that incur an error on the previous trial (one-tailed Z-test, Z-statistic: −23.25 (control), −5.87 (MCI) & −7.15 (SD), p < 0.001in each case). The log-odds ratio for correct trials between the Alzheimer and control groups is statistically lower than zero irrespective of the outcome of the previous trial (one-tailed Z-test, Z-statistic: 3.83 (Error − c), 3.46 (Error + c) & 4.46 (correct), p < 0.001 in each case). This indicates that the chance of incurring an error is higher for the AD group compared to the control group. A similar relationship is observed for the MCI group compared to the control (one-tailed Z-test, Z-statistic: 2.92 (Error − c), 1.79 (Error + c) & 8.13 (correct), p = 0.002, 0.037 & < 0.001 respectively).

### Did the probability of an error response *decrease* on the current trial, if the error on the previous trial was a corrected error (Error + c vs. Error − c)

An analysis was conducted to determine the potential impact of the Error + c trials in comparison to the Error − c trials. Table 2 reveals that there were a relatively lower proportion of errors following a corrected trial (Error + c) in comparison to an uncorrected trial (Error − c) for both the Alzheimer’s (Z = 2.954, p = 0.003) and MCI groups (Z = 3.297, p < 0.001). It is worth noting that this highly significant effect benefitted the MCI and AD groups rather than the healthy control group, precisely those clinical groups who generate uncorrected errors. This suggests that there was a level of adaptive control for the clinical groups who would benefit most.

Generalised Linear Multilevel Model Analyses (GLMM) were conducted to investigate the potential impact arising from participant variability. The fixed effects of the best model contained the cognitive groups and the outcome of the previous trial. Inclusion of an interaction between these was not significant (deviance test, deviance = 3.316, df: 4, p = 0.506). The inclusion of an age fixed effect was also not significant (deviance test = 2.798, df: 1, p = 0.094), indicating that the participant random effect captured the imbalance within the data. The error probability estimates (Fig. 1) confirmed the previous pattern of effects; the chance of an error AST trial increases across groups with increasing cognitive impairment. Moreover, the effect of a previously correct AST trial improves the chances of a correct behaviour on the current trial. The increased uncertainty in the probability estimates relate to the fact that the standard deviation of the random effect was estimated at 1.524 (bootstrap 95% confidence interval: 1.309–1.704). The inclusion of the random effect in the model is highly significant (deviance test, dev = 614.742, df: 1, p < 0.001); the estimated standard deviation is larger than all of the estimated coefficients (see Table 3). Despite being able to identify the key trial and group dependence structure, this high standard deviation estimate suggests that a large proportion of the variability within the data originates within the individual differences in the baseline scores in the ASTs. Yet, so far the analyses have only addressed the effects of the immediately preceding trial (trial n-1). The subsequent analyses address the potential effects on trials n-2, n-3, n-4 etc. on the current trial.

**Table 3:** GLMM fixed effect estimates for the dependence of antisaccade error trials (i.e. incorrect prosaccade) on the sequence length of previous correct or error trials.

Variable	Estimate (Log-odds)	Std. Error	Z value	P value
**Intercept**				
Control Group	0.741	0.164	4.534	<0.001
MCI Group	−0.200	0.189	−1.059	0.290
Alzheimer Group	−0.344	0.236	−1.455	0.146
**Slope**				
No. Previous Success	0.047	0.018	2.666	0.008
No. Previous Fail	−0.040	0.020	−2.028	0.043

Participant random effect standard deviance estimate is 1.374 (95% confidence interval: 1.175–1.543).

### Do the effects of the past outcomes in AST extend beyond the most recent trial? Are the effects of the previous error (or correct responses) cumulative

This section describes the effects of the sequence length on the correct and error trials.

**Figure 2:**
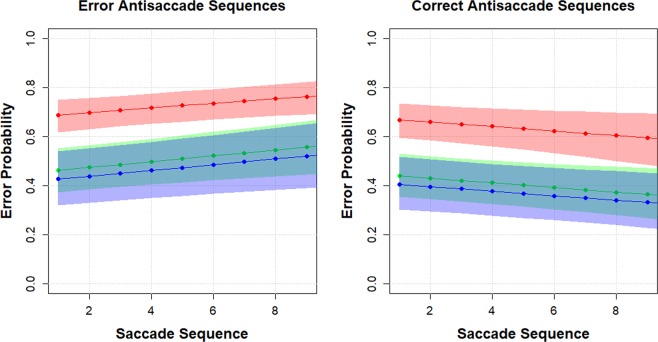
Predicted probability for an average participant in each group depending on the sequence length of the previous trials. Red = control; Green = Mild Cognitive Impairment; Blue = Alzheimer’s groups.

Figure 2 presents the predicted probability for an average participant in each of the groups depending on the sequence length of the previous trials. It shows the predicted probability of errors (with shaded 95% confidence intervals) as a function of the number of consecutive error and correct trials. If the sequence is of correct trials, then the probability that the current trial is an error decreases with the sequence length for all groups, whilst this error probability increases for all groups in the case of an increasing sequence length of error trials.

Table 4 presents the sample proportions for a correct current trial in relation to the sequence lengths. A significant deviation from the sequence length of 1 (i.e. dependence on the last trial only) was found for all groups, where the current error sample proportions decrease with the increasing sequences of correct trials whilst increasing for sequences of error trials. Subject variation was accounted for by fitting a GLMM.

**Table 4:** Proportion of error antisaccade trials, by participant group, for various lengths of immediately preceding and consecutive correct or error trials.

Length	Correct sequence			Error Sequence		
	Control	MCI	AD	Control	MCI	AD
1	0.354	0.504	0.461	0.436	0.521	0.588
2	0.300	0.439	0.462	0.510	0.582	0.532
3	0.279	0.314**	0.317	0.771**	0.683**	0.837**
4	0.130**	0.370	0.077**	0.765**	0.704*	0.825**
5	0.227*	0.192**	0.174*	0.795**	0.757**	0.800*
6+	0.101**	0.136**	0.153**	0.792**	0.893**	0.903**

Asterisk denotes proportions that are significantly different from the corresponding length 1 proportion at the 5% (*) and 1% (**) levels. (Two-tailed Z-test applied to the corresponding log-odds ratio estimates).

The optimal model, summarised in Table 4, contains cognitive group, and the number of previous correct and previous error trials as fixed effects. The effect of subject age was not significant (deviance test, deviance = 2.969, df: 1, p = 0.085), nor was there a significant interaction between the covariates (deviance test, deviance: 1.250, df: 4, p = 0.870). This provides no evidence that the slopes are significantly different between the groups. The inclusion of the random effect had a significant effect (deviance test, deviance: 259.024, df: 1, p = <0.001) with a participant standard deviation of is 1.374 (bootstrap 95% confidence interval: 1.175–1.543).

These analyses support a common principle that increases the probability of an error on the current trial. Past consecutive errors and correct responses predict future error and successes respectively. Apparently, the effect is cumulative. This principle applies to people with and without dementia. What distinguished the groups was not the principle, but the scaling of the effect. The higher the proportion of consecutive past errors, the more likely it is that the current trial will be an error. People with dementia commit more overall errors as a consequence of the disease. Importantly, the effects are already evident at the early MCI stage of the disease.

### Is there a common underlying mechanism for errors and correct antisaccades

Race models predict a strong relationship between the reaction time of the antisaccade response and the probability of a correct saccade^2,11^. Given that volitional saccades require a longer programming time, faster latencies will allow less time for the competing volitional saccade to be generated. Longer reaction times will increase the time for the volitional neural activation of the neural signal to reach the threshold for activating the saccade. If this process applies equally to the brain with and without dementia, then we would predict that the reaction time plots in relation to error and error responses would be isometric and overlapping, irrespective of the clinical condition. We will return to this point in the discussion section.

**Figure 3:**
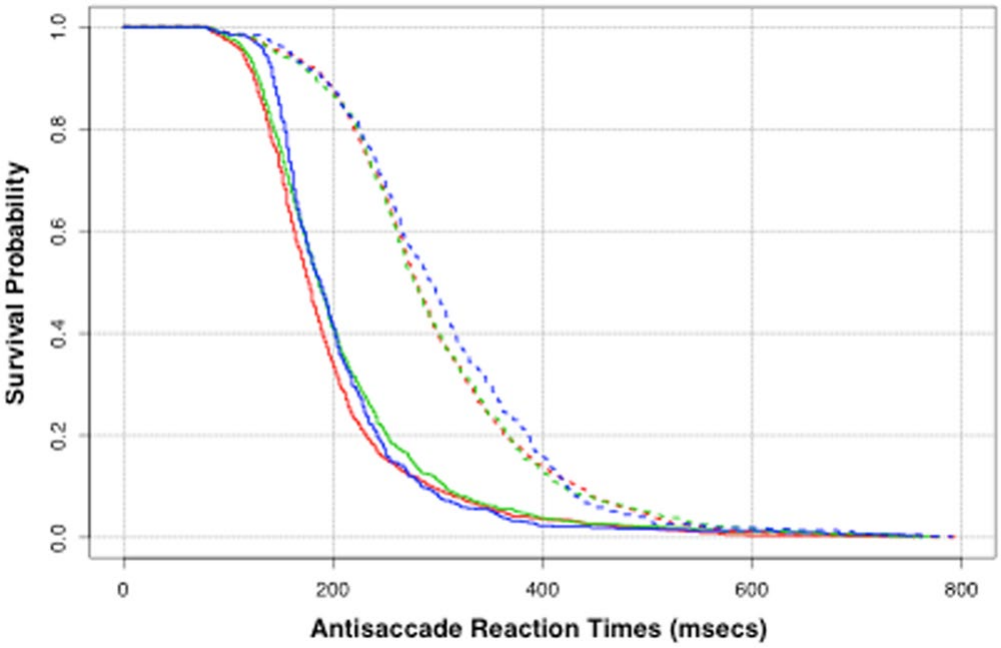
Kaplan-Meier plot of the reaction time survival curves. Dashed – correct antisaccade trials; Solid – Error antisaccade trials (irrespective of whether the error was corrected or not). Red - Control; Green – MC; Blue – Alzheimer.

Figure 3 presents the Kaplan-Meier plot of the survivor curves for participant reaction time. This describes the proportion of participants who are yet to record a saccadic event (correct saccade vs error saccade) at the given duration of time. It is immediately apparent that the trials that result in a correct antisaccade have longer reaction times than those trials with an error. Any differences in reaction time between cognitive groups are only subtle. As the distribution of reaction times was asymmetric, the responses were transformed onto the log scale prior to modelling. The difference in reaction time with regards to the trial outcome remained significant on this scale (Welch two sample t test, t = −37.454, df: 4364, p < 0.001; Cohen’s d = 1.118, R-squared = 0.243). Table 5 shows the median latencies and 95% confidence intervals for the correct and incorrect trials. The relative latency difference between correct and incorrect antisacades was remarkably similar across the groups (Controls = 104 ms; MCI = 92 ms; AD = 106 ms).

**Table 5:** Median reaction time and 95% confidence interval of the median estimate.

Initial event	Control	MCI	Alzheimer
Correct	280 (276–286)	278 (270–286)	294 (282–306)
Error	176 (172–180)	186 (182–192)	188 (180–194)

## Discussion

A growing number of studies have demonstrated that endogenous saccadic eye movements (i.e. antisaccades) are highly sensitive to the impairment of cognitive control in the AD group, and the reflects to some extent the dysfunction of working memory and inhibitory control^5,11,12^. However, this is the first study in AD to investigate the effects of the past trials on the behaviour of the current trial. We employed the AST to examine this, as this simple task is well tolerated by people with dementia and yields a frequency distribution of correct and error responses in patients and healthy controls. The following questions were under investigation: To what extent did an error (or correct saccade) on the previous trial influence the outcome on the current trial? Is there a cumulative effect of the past errors (or correct saccades) on the current behaviour? What is the relationship between AST errors and saccade reaction times? The findings were clear and compelling. A previous error or a consecutive sequence of errors increased the probability of an error on the current trial. Conversely, a correct saccade on the previous trial increased the probability of a correct response in the future. The effects were cumulative with longer sequences of errors (or corrections) predicting a proportionally higher probability of future errors (or corrections). The current findings are consistent with the previous work on healthy young adults, which reported a similar effect^2^ (Tatler & Hutton, 2006). Interestingly, the errors were significantly reduced on the trials that followed a previously corrected error, compared to trials where the error remained uncorrected, revealing a level of adaptive control in participants with early dementia. This indicates that participants with early (MCI) or a later stage of dementia (AD) retain a level of adaptive cognitive control that may help to modulate the errors. This effect was not significant for the control group when analysed in isolation, probably due to the well-known low level of uncorrected errors in healthy controls.

It is worth considering the potential factors that could have given rise to the cumulative effects of the AST errors. According to the race competition models of the AST a “race” commences, at the onset of the target, between the exogenously triggered prosaccade and the endogenous antisaccade signal^11^. The signal that first reaches the threshold “wins” the competition and triggers the saccade. The threshold of activation for either of the two competing signals can be modified by modulating the rate at which activation of the neural signal rises to the threshold, or the baseline level of activation before the stimulus onset^13–15^. Therefore, one possibility is that there is a persistent and stable increase in the baseline level of activation in the exogenous (i.e. prosaccade) and/or a decrease in the baseline of the endogenous (antisaccade) signal across the trials^6^ giving rise to the increased errors. In this way, with each additional error in the sequence, a growing tendency emerges towards further errors. Relatively fast reaction times are associated with the errors (i.e. prosaccade) in comparison to the correct, but slower antisaccade, as revealed in Fig. 3. Keeping in mind the fact that the frequency of the uncorrected errors was also greater in participants with dementia, this may be due to an additional problem with the internal monitoring of action that appears to be compromised in AD. This is in contrast to participants with Parkinson’s disease and schizophrenia , whose errors are usually associated with a subsequent rapid and spontaneous error correction^3,16^.

Another possible explanation can be derived from our work on the effects of glucose and motivation on antisaccades^17^. A number of studies have shown that individuals find it difficult to complete two consecutive self-control tasks that require cognitive effort; the first is completed as usual, but there is usually a decline in performance on the second task^18^. One influential theory^19^ states that this decline stems from a depletion in the limited resources of self-control, since the effect is specific to self-control tasks and not found for automatic tasks^18,20^. Since glucose was claimed to be the source of this critical energy, we hypothesised that a glucose supplement should be able to mitigate against this decline in self-control^21^. Our results did not support this theory. Using a double-blind study, we found that glucose did not protect the antisaccade performance following a Stroop task. However, there was a strong effect of the motivation level on the AST errors. High levels of motivation were associated with a low frequency of AST errors. Therefore, given that the motivation levels mediated the AST errors in the Kelly *et al*.^17^ study, it is conceivable in the current work that motivation levels may also have been associated with the frequency of errors. One can imagine a descending cascade where an error leads to the depletion of motivation, which then leads to a further error. This cascade could be a mediating factor in the accumulative effects of the AST errors. A consecutive sequence of successful correct saccades may have protected against the decline in task motivation, in contrast to a series of failures, which would be more likely to have fostered a lower level of motivation. Clearly, further work will be required to evaluate the validity of this hypothesis.

Inferences that are derived from the neurocognitive work on Alzheimer’s disease have often been contingent on a number of assumptions in relation to the participant’s performance on the cognitive task. For example, there is an assumption that the participant has a full understanding of the task and is sufficiently motivated to perform well. A major appeal of the saccadic eye movement paradigms is that these can be applied irrespective of a verbal or somatomotor dysfunction, and therefore can be used for the assessment of cognition across a wide range of domains including clinical, animal neurophysiological and infancy research^2,22^. The sessions are kept as short as possible to protect against the loss of concentration and motivation. We use a blocked presentation of trials design and we started each testing session with the AST to avoid the well-known priming effects from the prosaccade task. Note also the selective nature of the eye-tracking impairments in AD. For example, participants with AD dementia performed remarkably similar to controls in an attentional disengagement saccade overlap task^23^ which demonstrates that prosaccadic eye movement paradigms are unlikely to be inherently cognitively demanding for these participants. This increases our confidence that the patients understood the antisaccade task and were motivated to perform well, given the evidence that on a high proportion of the trials, the errors were self-corrected.

In considering the impairment of saccadic eye movements in AD group it is important highlight again its selective nature given that a number of keys features were in fact, well preserved. For example, prosaccade amplitudes, peak velocity and the attentional disengagement (measured by the GAP effect) were preserved and remain stable over time^3,23^. The current work revealed that inhibitory control in AD was influenced by the past performance in a similar fashion to the healthy controls. Similarly, errors were associated with faster reaction times in a similar manner to the control group. These findings revealed that there is no fundamental change in the principles that govern the regulation of AST errors in people with dementia. Apparently, the dementia status adds a positive constant to the AST errors but there is a common underlying principle in relation to the effect of past error and success on the current behaviour.

## Methods and Materials

Two hundred and two participants were included in this study: 42 with dementia due to Alzheimer’s disease (AD) and 65 with mild cognitive impairment (MCI); Control participants were 95 volunteers who were either the spouses of the AD or MCI participants or were research volunteers from the North West of the UK. Participants with dementia were referred and diagnosed via memory clinics in the United Kingdom National Health Service after a comprehensive evaluation by the dementia assessment team. The participants were all white British or European with a minimum of 11 years of education and fluent in the English Language (age and gender characteristics are shown in Table 1). Participants with a diagnosis of dementia due to AD satisfied the NINCDS-ADRDA criteria^24^. The following criteria were required for the diagnosis of MCI^25^: (1) a subjective complaint of a decline in memory function (that was confirmed by the individual or his/her informant/primary care giver); (2) an objective impairment using a formal memory test or other cognitive impairment (where the scores on the standard cognitive tests were at least 1.5 SDs below the age-adjusted adjusted norms); (3) a relative preservation in the activities of daily living. The following exclusion criteria were applied: a previous history of head trauma, stroke, cardiovascular disease, active or past alcohol or substance misuse/dependence, or any physical or mental condition severe enough to interfere with their ability to participate in the study. All participants had the capacity to consent to participation in the study and gave signed informed consent. The study received ethical approval from the local NHS Research Ethics Committee, NHS Health Research Authority and Lancashire Care Teaching Hospital Trust. These experiments were performed in accordance with the UK British Psychological Society ethical guidelines.

### Stimuli and Tasks

#### Apparatus

Eye movements were recorded using the EyeLink Desktop 1000 eye-tracker (SR Research) sampling at 500 Hz. Participants were seated 55 cm away from the display monitor (60 Hz). The Miles test (Roth, Lora and Heilman (2002)) was used to determine the dominant eye and tracked accordingly. The raw data was exported from the EyeLink to the DataViewer software and analysed offline with bespoke software^26^. To filter noise and spikes all frames were removed where the velocity signal was greater than 1,500 deg/s or the acceleration signal was greater than 100,000 deg^2^/sec. The latency of the saccade was measured from the onset of the saccade to the target onset. Fixations and saccadic events were detected using the EyeLink parser which filtered out anticipatory saccades (i.e. saccades which were programmed before the presentation of the distractor) and excessively delayed saccades (due to inattention) using a temporal window of 80–700 ms measured from the onset of the target.

#### Antisaccade task (AST)

**Figure 4:**
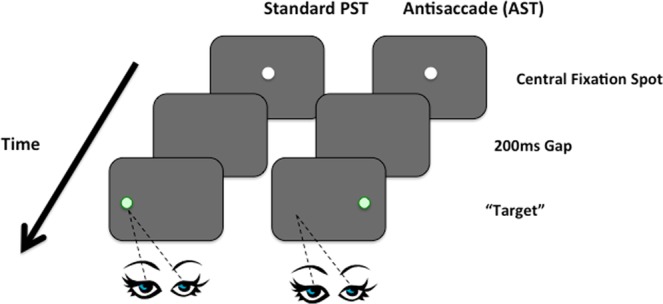
Standard prosaccade task (PST) showing the eye directed towards the green target. In the antisaccade task (AST) the eye is directed away from the target to the opposite hemifield.

Each trial was preceded by a 1 second instruction screen stating that the participant should look away from the target. A central fixation target was displayed in white on a black background. This was displayed for one second. There was a 200 ms blank interval before the appearance of the saccade target. The saccade target (in red) was then presented in a random order 4 degrees away from the fixation target on the left or right side for 2 seconds. Participants were asked to fixate on the central fixation spot and then to generate the saccade to the opposite side of the screen as soon as the target appeared on the display (Fig. 4). We introduced several procedures to increase the compliance of the dementia groups. Each trial was preceded by an instruction screen that clearly explained to the participant how to direct the eyes on an antisaccade trial. Extensive pilot work in our lab has revealed that participants with AD can tolerate a limited number of trials without loss of concentration and mental fatigue. Therefore, in order to reduce the effects of experimental fatigue we restricted the AST to 24 trials.

#### Cognitive assessment

The Dementia Assessment: Montreal Cognitive Assessment^27^ is a brief screening tool for Alzheimer’s dementia that includes the cognitive functions of attention and concentration, executive control, memory, language, visuo-constructional skill, conceptual thinking, calculation and orientation. A score between 0–25 (max = 30) is considered to be within the dementia range.

The digit span task^28^ assesses phonological memory function; participants were presented verbally with a set of single digit numbers and required to recall the digits in the correct serial order. The number of digits gradually increased during the course of the experiment, starting with two and going up to a maximum of eight. Two trials were presented at each level of difficulty and a participant must get both correct in order to progress to the next level. Participants were then presented with a reverse digit span task, where they were required to name the items in the reverse order of the presented sequence. Again, the number of digits were presented incrementally, from two to eight. Two trials were presented at each level, both needed to be correct in order for the participant to progress. A total digit span score was then calculated out of 16 for each of the forward and reverse digit spans tests.

In the spatial span task^29^ participants were presented with a board containing an irregular array of nine square blocks. The experimenter pointed to each block, one block at a time in a given pseudo-randomised sequence. At the end of the sequence, the participant was required to point to each of the blocks that were indicated in the correct serial order. The number of square blocks selected in a sequence was increased over the course of the experiment, from two up to a maximum of eight. Two trials were presented at each level and the participant had to achieve both correct in order to progress to the next level. A reverse version of the task was also conducted with the participant indicating the sequence in the reverse order. A total spatial span score was then calculated out of 16 for each of the forward and reverse forms of the tests.

### Statistical analysis

The principal aim of these analyses was to search for any evidence that the outcome of the current trial (error vs. success) and/or the reaction times was predicated on the outcome of previous trial(s), the participant’s cognitive grouping or another explanatory variable. It is important to take into account each participant’s predisposition for a particular outcome. Any given set of participants may have very different natural error rates irrespective of their group membership or demographic status. Accounting for specific effects at the level of each individual recognises that the trials are nested within a participant, and consequently enables a better quantification of the main effects of interest. This was achieved using multilevel models, also known as hierarchical linear model or random coefficient models^30–32^.

Two dependent variables for the eye-tracking tasks were entered into these analyses: the trial outcome (i.e. whether or not an inhibitory error was committed in the AST: error or correct); and the saccade reaction times after the appearance of the “distractor” for the errors and the correct responses. Let y_i,j_ denote the trial outcome for participant i at trial j, which describes a Bernoulli trial that takes the value 1 for an inhibitory control error and 0 for a correct saccade. The trial probability for an AST error p_i,j_, is mapped to the real-line using the logit transform, ${{\rm{\mu }}}_{{\rm{i}},{\rm{j}}}^{({\rm{y}})}={\rm{logit}}({{\rm{p}}}_{{\rm{i}},{\rm{j}}})$, which is then characterised by the linear predictor of explanatory variables given in Eq. (1) below. For the second response, we denote t_i,j_ as the log of the reaction time for participant i at trial j. The reaction times are transformed onto the log-scale to reduce the asymmetry in the measurements. These measurements can be described using the linear regression model ${{\rm{t}}}_{{\rm{i}},{\rm{j}}}={{\rm{\mu }}}_{{\rm{i}},{\rm{j}}}^{({\rm{t}})}+{{\rm{e}}}_{{\rm{i}},{\rm{j}}}$ where the predictor, ${{\rm{\mu }}}_{{\rm{i}},{\rm{j}}}^{({\rm{t}})}$, is a linear function of explanatory variables (Eq. (1)) and e_i,j_ is the residual error associated with each observation. The predictors ${{\rm{\mu }}}_{{\rm{i}},{\rm{j}}}^{({\rm{y}})}$ and ${{\rm{\mu }}}_{{\rm{i}},{\rm{j}}}^{({\rm{t}})}$ are linear functions of the explanatory variables and the participant specific effects. In general, the linear predictor is defined to be:

1 $${\mu }_{i,j}={\beta }_{0}+{u}_{i}+{\beta }_{1}{x}_{1,i,j}+{\beta }_{2}{x}_{2,i,j}+\ldots $$

where ${{\rm{x}}}_{1,{\rm{i}},{\rm{j}}},\,{{\rm{x}}}_{2,{\rm{i}},{\rm{j}}},\ldots$ denote the explanatory variables (e.g. cognitive group, age, gender, last trial outcome, etc.) and ${{\rm{\beta }}}_{0},{{\rm{\beta }}}_{1},{{\rm{\beta }}}_{2},\ldots$ are the co-efficients of the main effect that are to be estimated. The decision on which explanatory variables were significant and included into each model was determined by using a backwards-variable selection with the deviance hypothesis test. The random quantity u_i_ represents participant i’s deviation from the overall baseline value, β_0_. These subject effects are assumed to follow a zero-mean normal distribution with unknown variance to be estimated. All of the analyses were conducted in R statistical software^33^ using the “lme4” package^34,35^ for inference for the linear and generalised linear multilevel models (GLMM). The additional package “survival”^36,37^ was used to evaluate and summarise the reaction time survival curves in Fig. 3.

## Data Availability

The data supporting the conclusions of this manuscript will be made available by the authors, without undue reservation, to any qualified researcher on reasonable request.
